# Evaluating the protective efficacy of a trivalent vaccine containing Akabane virus, Aino virus and Chuzan virus against Schmallenberg virus infection

**DOI:** 10.1186/1297-9716-44-114

**Published:** 2013-12-05

**Authors:** Silke Hechinger, Kerstin Wernike, Martin Beer

**Affiliations:** 1 Institute of Diagnostic Virology, Friedrich-Loeffler-Institut (FLI), Suedufer 10, 17493 Greifswald, Insel Riems, Germany

## Abstract

Schmallenberg virus (SBV), an arthropod borne pathogen, spread rapidly throughout the majority of Europe since 2011. It can cause a febrile disease, milk drop, diarrhea, and fetal malformation in ruminants. SBV, a member of the Simbu serogroup within the genus *Orthobunyavirus*, is closely related to *Akabane virus* (AKAV) and *Aino virus* (AINOV) among others. In the present study, 4 Holstein-Friesian calves were immunized twice four weeks apart with a multivalent, inactivated vaccine against AKAV and AINOV. Another 4 calves were kept as unvaccinated controls. All animals were clinically, serologically and virologically examined before and after challenge infection with SBV. AKAV- and AINOV-specific neutralizing antibodies were detected one week before challenge infection, while SBV-specific antibodies were detectable only thereafter. SBV genome was detected in all vaccinated animals and 3 out of 4 controls in serum samples taken after challenge infection. In conclusion, the investigated vaccine was not able to prevent an SBV-infection. Thus, vaccines for other related Simbu serogroup viruses can not substitute SBV-specific vaccines as an instrument for disease control.

## Introduction

Schmallenberg virus (SBV), a member of the Simbu serogroup within the genus *Orthobunyavirus*, family *Bunyaviridae*, emerged in Europe in autumn 2011 [1]. Thereafter it spread rapidly throughout large parts of the continent [2]. Blood sucking insects, particularly midges of the *Culicoides obsoletus complex*, are involved in the transmission of the pathogen [3-5]. In adult cattle, sheep and goats mild febrile disease accompanied by reduction of milk yield may be observed, sometimes associated with diarrhea [6]; inapparent infection may occur as well. Fetal infection during a critical phase of pregnancy may lead to damage of the central nervous system and the musculoskeletal structures [7,8]. Stillbirth or birth of weak calves, lambs or kids, abortion and dystocia are the possible consequences [6,9]. Experimental infection of cattle resulted in RNAemia for a few days and infection of diverse tissues throughout the body of the host [1,10].

SBV is closely related to *Sathuperi virus* (SATV) and *Douglas virus* (DOUV) [11,12], and it was demonstrated that SBV-specific antibodies are able to neutralize infectivity of DOUV, SATV and *Aino virus* (AINOV) in vitro [11]. Additionally, serological cross-reactions between *Akabane virus* (AKAV), AINOV, DOUV, SATV and *Shamonda virus* (SHAV) were described previously [13]. These cross-reactions were detected in complement fixation tests (CFT), not in neutralization tests. Consequently, the contribution of such antibodies to a protective effect might be limited. Beyond that, AINOV and AKAV cause symptoms in ruminants which are similar to those of an SBV-infection [14,15], and vaccines have been developed for disease control [16,17]. *Chuzan virus* (CHUV, family *Reoviridae*, genus *Orbivirus*) is another teratogenic pathogen of ruminants which occurs in Asia [18,19]; it has been included into a multivalent vaccine together with AKAV and AINOV. In the present study, the possible cross-protection of this multivalent vaccine against a subsequent challenge infection with SBV was investigated.

## Materials and methods

### Experimental design

The experimental protocol was reviewed by a state ethics commission and has been approved by the competent authority (State Office for Agriculture, Food Safety and Fisheries of Mecklenburg-Vorpommern, Rostock, Germany, ref. LALLF M-V TSD/7221.3-1.1-004/12).

Eight SBV-naive female Holstein-Friesian calves were divided in 2 groups of 4 individuals. The average age was 9.4 months at the first vaccination. The animals were housed under BSL 3 conditions during the entire study to prevent a natural SBV-infection.

Animals of group 1 (C01-C04) were immunized intramuscularly twice 4 weeks apart with 3 mL of a trivalent inactivated vaccine for AKAV, AINOV and CHUV (Nisseiken Bovine Abnormal Parturition Trivalent Inactivated Vaccine, Nisseiken Co., Ltd, Japan). The efficacy and safety of this vaccine have been investigated previously [16]. The second group (C05-C08) was used as unvaccinated control. Injection sites were monitored daily for 4 days after both vaccinations.

Six weeks after the first vaccination all animals were inoculated subcutaneously with 2 × 0.5 mL of an SBV field strain that was only passaged in cattle [10]. After the challenge infection the animals were monitored for clinical signs by veterinarians for eight days.

Rectal body temperature was recorded daily. Blood samples were collected weekly, starting from day 7 after the first vaccination (7 days post vaccination (dpv)), as well as daily on the 8 days following challenge. Serum samples were analyzed with a commercially available SBV antibody ELISA (ID Screen^®^ Schmallenberg virus Indirect, IDvet, France) and in standard microneutralization tests (SNT) against SBV, AKAV and AINOV [20].

Samples of spleen, tonsils, and mesenteric and mandibular lymph nodes were taken at autopsy and homogenized in 1 mL of Minimum Essential Medium (MEM).

### RNA extraction and real-time RT-PCR

RNA was extracted from serum and tissue samples using the MagAttract Virus Mini M48 Kit (Qiagen, Germany) according to the manufacturer’s recommendations.

SBV genome load was determined by an SBV-specific reverse transcription real-time PCR (real-time RT-PCR) as described previously [21] with an external standard based on the small (S) genome segment.

## Results

### Clinical observation and pathology

None of the animals showed any signs of clinical disease. Body temperatures were within a normal range for all animals. The measured temperatures never exceeded 39.5 °C. Additionally, no adverse side effects were observed following either vaccination. Autopsy did not reveal any significant gross lesions.

### Serology

**Figure 1:**
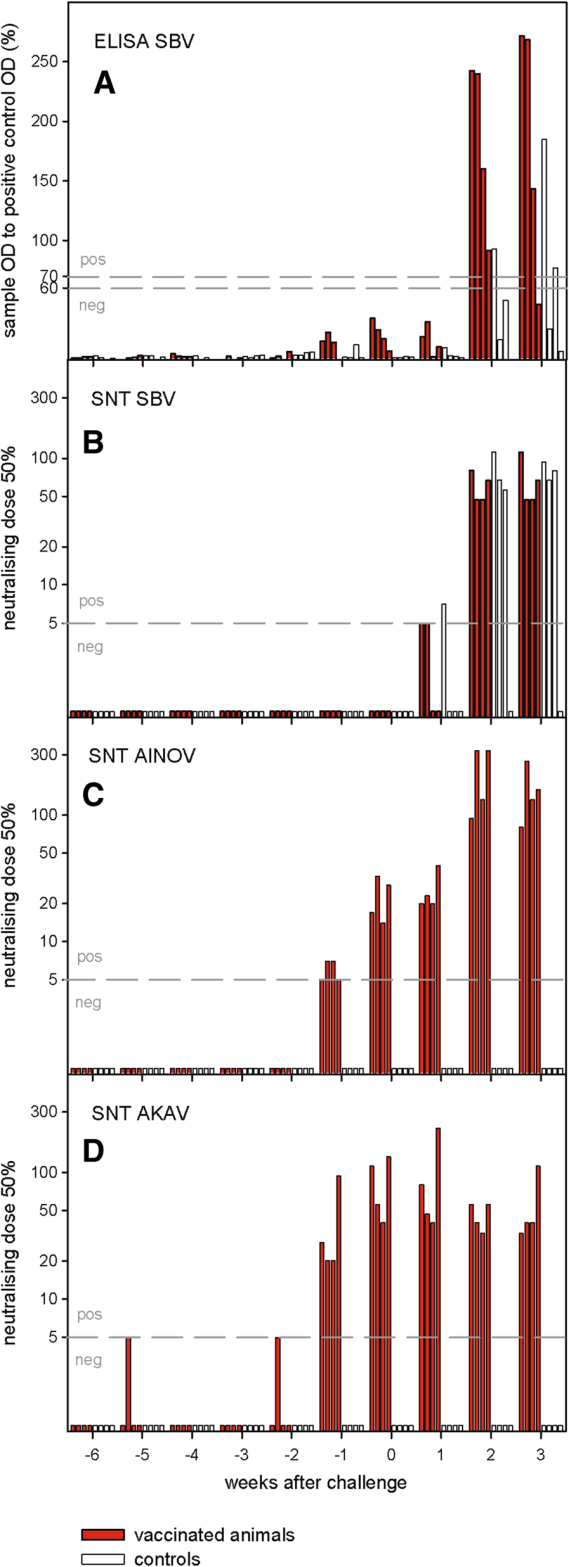
**Serology.** The animals were vaccinated 6 and 2 weeks before challenge. Bars represent one animal each. Serum samples were tested by a commercially available SBV antibody ELISA **(A)** and in standard microneutralization tests against SBV **(B)**, AINO **(C)** and AKAV **(D)**. Horizontal dashed lines indicate the cut-off value of the respective test. The neutralization titers are expressed as reciprocal of the serum dilution showing 50% virus neutralization.

All animals were seronegative for SBV, AINOV and AKAV before first vaccination (Figure 1).

In one vaccinated animal first AKAV-specific antibodies could be detected one week after the first vaccination and two weeks prior to challenge, respectively. All immunized animals were SNT-positive for AKAV and AINOV one week before challenge infection. First SBV-specific neutralizing antibodies were detected in two of four animals one week after challenge (Figure 1). Two weeks after challenge infection all four vaccinated animals were positive for SBV, both in SNT and ELISA.

The four control animals remained seronegative for AKAV- and AINOV throughout the study. SBV-specific antibodies were detected starting from the second week after challenge infection, with three animals being positive in the SNT, and one animal scoring positive in the ELISA. A second control animal showed a positive reaction in the ELISA in the third week after challenge infection. One control animal (C05) remained negative for SBV in both serological assays until the end of the study.

### Real-time RT-PCR

**Figure 2:**
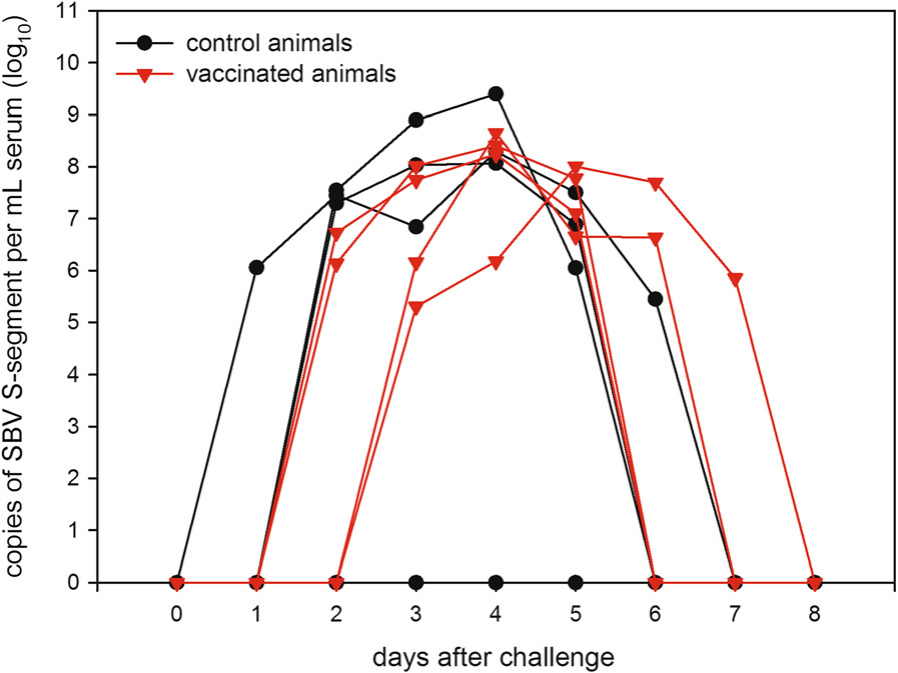
**Real-time RT-PCR results for serum samples after challenge infection.** All vaccinated animals (depicted in red) and three out of four control cattle (black) scored positive in the RT-qPCR for several days.

All vaccinated animals as well as three of four control animals were positive in the RT-PCR (Figure 2). SBV genome was detected in the serum samples of all vaccinated animals and in all control animals with the exception of C05. In group 1 (vaccinated animals) RNAemia was seen from 2 days post challenge (dpc) infection to 5 dpc in two animals (C03, C04), from 3 dpc to 6 dpc in one animal (C02) and from 3 dpc to 7 dpc in the remaining animal (C01). In group 2 (controls), the animals showed RNAemia from 2 dpc to 5 dpc (C06, C08) and from 1 dpc to 6 dpc (C07), respectively.

Furthermore, SBV genome was detected in all vaccinated animals in the mesenteric lymph nodes (average: 3.89 × 10^5^ genome copies/mg sample material), the spleen samples (average: 1.01 × 10^5^ copies/mg) and mandibular lymph nodes (average: 3.59 × 10^4^ copies/mg). In tonsils of C01 and C03 1.55 × 10^5^ copies/mg and 1.82 × 10^2^ copies/mg were detected.

In control animals C06, C07 and C08, SBV genome was detected in mesenteric lymph nodes (average: 1.66 × 10^6^ copies/mg) and spleens (average: 3.05 × 10^5^ copies/mg). In C07 and C08 mandibular lymph nodes were positive as well (1.36 × 10^6^ copies/mg and 5.08 × 10^5^ copies/mg respectively) and tonsils of C07 contained 4.92 × 10^2^ copies/mg. In samples from calf C05 SBV-genome was not detectable.

## Discussion

Antibodies specific for Simbu serogroup viruses frequently cross-react with more than one other member of this serogroup [11,13]. Such interaction might also influence viral replication in vivo. After the emergence of SBV in Europe, vaccines against related Simbu viruses, such as AKAV and AINOV, could potentially offer a tool for disease control until an SBV-specific vaccine is ready for use.

Considering the current epidemiological situation vaccination of young female sheep or cattle before their first pregnancy will be an important measure to eliminate the risk of SBV-infection of naïve animals during the vulnerable phase of fetal development. Therefore, calves instead of cows were used in the present study. Data about the effect of vaccination of calves were not published for the vaccine we used but the efficacy of another inactivated AKAV-specific vaccine in calves aged 5 to 10 months has been proven [17]. Additionally, inactivated SBV vaccines are efficacious in calves [22].

The trivalent AKAV/AINOV/CHUV-vaccine applied in the present study has proven its effectiveness [16]. Neutralizing antibodies against AINOV and AKAV could be detected shortly after the second vaccination. For AKAV it has been demonstrated, that a neutralizing titer of 16 in experimental animals prevented RNAemia after infection in comparison to one control animal [16]. Thus, we assume that a mean neutralizing titer of 85 (minimum 40), as detected in the present study, would have provided protection against AKAV infection. For AINOV protective antibody titers could not be determined from the literature. However, the titers detected in the present study are comparable to or even exceeded those given for cattle in earlier studies on this vaccine [16]. Therefore, protection could probably have been expected in case of an AINOV-infection also.

SBV-specific antibodies were detectable only after challenge infection. In the ELISA some low activity was seen for the samples of vaccinated animals in week -1 and 0 (Figure 1). At the same time anti-AKAV and anti-AINOV antibodies started to be detectable in vaccinated animals by neutralization test. Serological cross-reaction between close relatives of SBV (SATV, DOUV, SHAV) and AKAV/AINOV have already been described in CFT [13]. This test also detects non-neutralizing antibodies like anti-nucleoprotein antibodies. As the ELISA that was used for our analysis is based on recombinant SBV nucleoprotein for antibody detection this can explain the results.

Unlike Goller et al. [11] we did not detect cross-neutralization between AINOV and SBV. One possible reason for this discrepancy is the determination of the neutralizing activity of anti-AINOV/AKAV antibodies towards SBV in the present study, but of neutralizing activity of anti-SBV antibodies towards AINOV and other Simbu viruses by Goller et al. [11]. In contrast to the nucleoprotein-based ELISA, neutralization depends on antibodies binding to viral glycoproteins. This can explain that there was some evidence of cross reactivity in the ELISA but not in the neutralization test. Furthermore, the AINOV/AKAV antibodies in our study were induced by vaccination while the SBV-antiserum used by Goller et al. [11] was collected following SBV-infection.

After inoculation with SBV, viral RNA was present in serum samples of all vaccinated animals for several days and in 7 of 8 animals SBV-RNA was detected in the lymphatic tissues sampled at autopsy. The same was observed in unvaccinated control animals during SBV vaccine studies, whereas vaccination with inactivated prototype SBV vaccines has been associated with reduced RNA load in serum and tissue samples or no detection of SBV genome at all, even if SBV-specific antibody titers were low [22].

Remarkably, highest SBV-genome loads for tissue samples were found in mesenteric lymph nodes in most animals. This is in agreement with results from earlier studies [10]. However, the role of lymphatic tissues in the pathogenesis of SBV infection has not been thoroughly investigated so far and is a topic to which attention should be paid in future research.

It is unknown why one animal (C05) failed to show signs of infection both in serological tests and PCR but similar observations have been made after experimental SBV-infection of sheep [23]. One explanation could be a failed injection, another one a general resistance to SBV-infection of unknown cause.

In conclusion, protection against SBV-infection could not be proven for the multivalent vaccine tested. Thus, vaccines for other related Simbu serogroup viruses can not substitute SBV-specific vaccines as an instrument for disease control.

## Competing interests

The authors declare that they have no competing interests.

## Authors’ contributions

Conceived and designed the experiments: KW, MB. Performed the experiments: SH, KW. Analyzed the data: SH, KW. Wrote the paper: SH, KW, MB. All authors read and approved the final manuscript.
